# Somatic *PRDM2* c.4467delA mutations in colorectal cancers control histone methylation and tumor growth

**DOI:** 10.18632/oncotarget.21713

**Published:** 2017-10-09

**Authors:** Tatjana Pandzic, Veronica Rendo, Jinyeong Lim, Chatarina Larsson, Jimmy Larsson, Ivaylo Stoimenov, Snehangshu Kundu, Muhammad Akhtar Ali, Mats Hellström, Liqun He, Anders M. Lindroth, Tobias Sjöblom

**Affiliations:** ^1^ Science for Life Laboratory, Department of Immunology, Genetics and Pathology, Uppsala University, Uppsala, Sweden; ^2^ Department of Cancer Biomedical Science, National Cancer Center Graduate School of Cancer Science and Policy, Goyang-si, Republic of Korea

**Keywords:** colorectal cancer, PRDM2, genome editing

## Abstract

The chromatin modifier *PRDM2/RIZ1* is inactivated by mutation in several forms of cancer and is a putative tumor suppressor gene. Frameshift mutations in the C-terminal region of *PRDM2*, affecting (A)8 or (A)9 repeats within exon 8, are found in one third of colorectal cancers with microsatellite instability, but the contribution of these mutations to colorectal tumorigenesis is unknown. To model somatic mutations in microsatellite unstable tumors, we devised a general approach to perform genome editing while stabilizing the mutated nucleotide repeat. We then engineered isogenic cell systems where the *PRDM2* c.4467delA mutation in human HCT116 colorectal cancer cells was corrected to wild-type by genome editing. Restored *PRDM2* increased global histone 3 lysine 9 dimethylation and reduced migration, anchorage-independent growth and tumor growth *in vivo*. Gene set enrichment analysis revealed regulation of several hallmark cancer pathways, particularly of epithelial-to-mesenchymal transition (EMT), with VIM being the most significantly regulated gene. These observations provide direct evidence that *PRDM2* c.4467delA is a driver mutation in colorectal cancer and confirms *PRDM2* as a cancer gene, pointing to regulation of EMT as a central aspect of its tumor suppressive action.

## INTRODUCTION

Almost 15% of colorectal cancers (CRCs) have a defective DNA mismatch repair (MMR) system as a result of inactivation of an MMR gene (*MLH1, MSH2, MSH6* or *PMS2*) through mutations, deletions or epigenetic silencing [1]. In the absence of MMR, nucleotide alterations accumulate throughout the genome, particularly in short tandem DNA repeats (microsatellites). To date, more than 150 genes with somatic mutations within repeat tracts have been reported in cancers with microsatellite instability (MSI) [2–4]. While the majority of such mutations lack functional significance, a subset promote tumor cell growth and are therefore positively selected during the course of cancer development. A present challenge lies in assigning the contribution of MSI target genes to the pathogenesis of CRC and identifying those having a driver role [3]. High mutation prevalence is indicative of positive selection and is therefore used to identify driver genes [5, 6]. However, the high background mutation rate in MSI tumors, together with possible effects of the surrounding sequence may lead to increased mutation prevalence in certain coding repeats regardless of their role in carcinogenesis [7]. Hence, the ultimate proof of cancer gene status must come from functional studies that directly compare the mutant and wild-type alleles in relevant human cell systems.

The PR domain zinc finger protein 2 (*PRDM2*) gene, a member of a histone/protein methyltransferase superfamily that methylates Lys-9 of histone H3 through its N-terminal PR domain [8], is one of the frequently mutated genes in MSI CRCs. Two PRDM2 protein isoforms are produced through alternative promoters, with RIZ1 containing the PR domain and the methyltransferase-deficient RIZ2 lacking this domain [9]. Loss of 1p36, epigenetic silencing or inactivating mutations within the *PRDM2* PR domain are observed in many cancer types [8, 10, 11]. *PRDM2/RIZ1* knockout mice show increased susceptibility to a broad spectrum of tumors [12] whereas forced expression of PRDM2/RIZ1 suppresses tumorigenicity in nude mice [13]. These observations collectively support that *PRDM2* is a tumor suppressor gene, but the precise molecular mechanisms of its action have not been revealed to date and no consensus set of PRDM2 regulated genes has been identified. Frameshift deletion mutations within one of the two coding mononucleotide (A)8 or (A)9 repeats in exon 8 of *PRDM2* give rise to a truncated protein lacking the C-terminal PR-binding motif. These mutations are enriched in cancers exhibiting MSI with the highest prevalence observed in CRCs (~30% of MSI positive CRCs; [3]), gastric cancers (up to 50% of MSI positive cases) and endometrial cancers (up to 30% of MSI positive cases) [14]. *In vitro* characterization of truncation mutants has demonstrated the importance of the PR-binding motif in regulation of PRDM2 activity through PR binding and intra- and intermolecular interactions [9]. Direct evidence for its role in regulating PRDM2 function was provided recently by the demonstration that the C-terminus is necessary for H3K9 methyltransferase activity involved in establishing the H4K20me1-H3K9me1 *trans*-tail histone code at specific loci [15]. Despite their high prevalence the functional significance of *PRDM2* C-terminal truncating mutations in CRC and cancer in general has not been clarified. We therefore sought to identify the direct contribution of these mutations to carcinogenesis by restoring wild type-gene sequence of one mutant c.4467delA *PRDM2* allele in homozygous mutant CRC cells.

## RESULTS

### A general approach to correction of indel mutations in CRC cells with mismatch repair deficiency by repeat stabilization

Repeat tracts that are prone to acquire somatic mutation in MSI CRCs constitute challenging targets for genome editing and may be subject to reversal mutations after correction as a consequence of MSI. To avoid such reversion by re-mutation of corrected repeat sequences, we reasoned that long mononucleotide repeats could be divided into two shorter parts and thereby stabilized. This can be achieved by switching to an alternative codon encoding the same amino acid as the wild-type sequence while engineering base insertions. To determine general applicability, we analyzed genes known to harbour prevalent repeat mutations in CRC for the possibility to design recombinant adeno-associated virus (rAAV) knock-in constructs for repeat correction [16, 17] (Supplementary Table 2). Of the 25 repeats attempted, 18 were amenable to design of rAAV knock-in constructs, demonstrating the potential of this approach.

### Genome editing restores PRDM2 gene function in HCT116 human colorectal cancer cells

**Figure 1:**
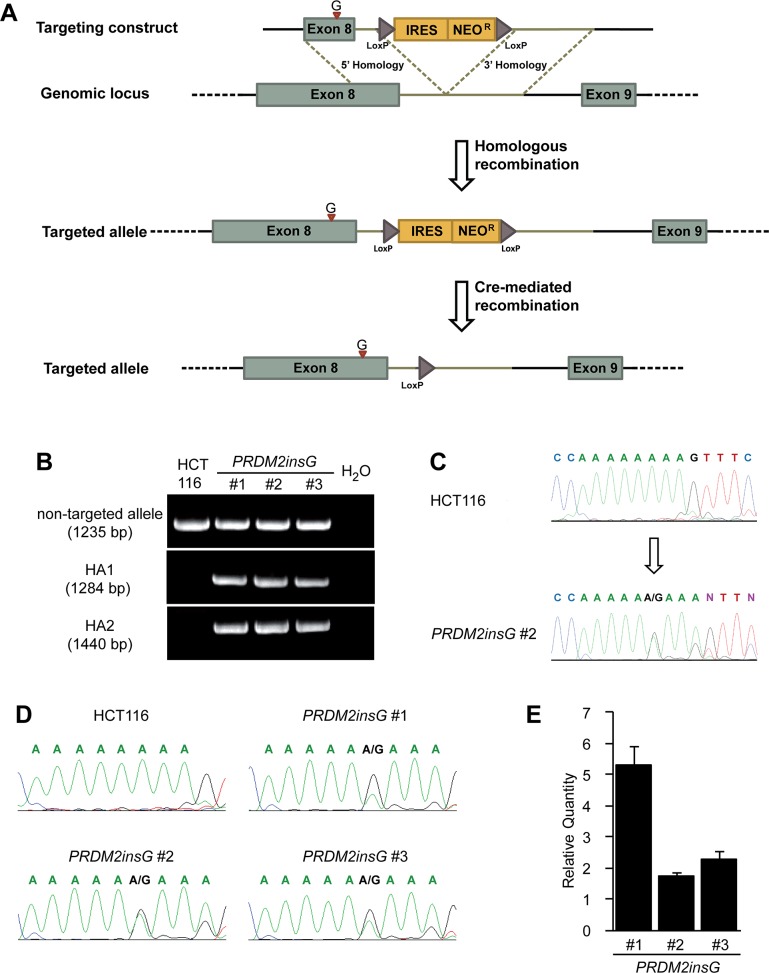
Restoration of endogenous *PRDM2* in HCT116 colorectal cancer cells **(A)** A rAAV gene targeting construct was designed to correct the c.4467delA mutation in one allele of *PRDM2* by introducing a base G at the position c.4464 within exon 8. The position of insertion is indicated with a red arrowhead. The construct also contains a promoterless selectable marker (IRES Neo) and LoxP sites that mediate the excision of the selection cassette. **(B)** Integration of the construct was identified by PCR. The upper panel shows the presence of wild-type allele in three *PRDM2insG* clones and parental HCT116 cells. The two lower panels show the presence of both homology arms (HA1 and HA2) in the targeted allele in *PRDM2insG* clones only. **(C)** Sequence analysis of gDNA demonstrates that the open reading frame of *PRDM2* is restored by inserting a base G into the truncated (A)9 tract (arrow, inserted base). Sequence chromatograms of parental HCT116 cells and *PRDM2insG* clone 2 are shown. **(D)** Sequence analysis of RT-PCR products confirming expression of the targeted allele. **(E)** RT-qPCR analysis of PRDM2 in three *PRDM2insG* clones demonstrates upregulation of PRDM2 after correction of c.4467delA in all three clones. Values are normalized to PRDM2 in parental HCT116 cells.

Frameshift mutations within coding C-terminal mono-nucleotide (A)8 and (A)9 repeats give rise to truncated PRDM2 lacking the PR binding motif. To understand the effect of these mutations, a rAAV mediated gene targeting system was used to generate isogenic cell lines where the only difference lies in the mutation of interest. The colorectal cancer cell line HCT116 was selected because it contains a homozygous c.4467delA mutation within the (A)9 repeat ([18] and this study). Bearing in mind its proposed tumor suppressor role, correction of one *PRDM2* allele in HCT116 should be sufficient to restore the PRDM2 protein function that is lost due to truncation. In search for additional cell models we genotyped the (A)9 repeat in 14 additional MSI+ CRC cell lines (Supplementary Table 4). We were not able to identify additional cell lines with homozygous c.4467delA mutations and therefore proceeded with genome editing of HCT116. The targeting vector was designed to insert one G at position c.4464 (codon 1487), correcting the reading frame and restoring wild type protein coding sequence while dividing the (A)9 tract into two shorter and more stable repeats (Figure 1A). PCR-based screening of gDNA confirmed the desired gene targeting event in 3 of 252 (1.2%) analysed clones (Figure 1B). The presence of *PRDM2insG* (c.4464insG) was verified by gDNA sequencing of the three targeted clones (Figure 1C). Direct sequencing of the RT-PCR product after the removal of the Neo resistance cassette by Cre recombination (Supplementary Figure 1) showed a heterozygous inserted G in all three clones, demonstrating transcription of the restored allele (Figure 1D). While the lack of reliable PRDM2 antibodies precluded protein expression analyses, the 1.7 to 5-fold expression increase observed in all clones after *PRDM2* correction (Figure 1E) combined with previous observations that mutant PRDM2 transcripts are eliminated by nonsense-mediated degradation [3] provided support for correct gene targeting.

### Effects of restored PRDM2 function on H3K9 methylation

**Figure 2:**
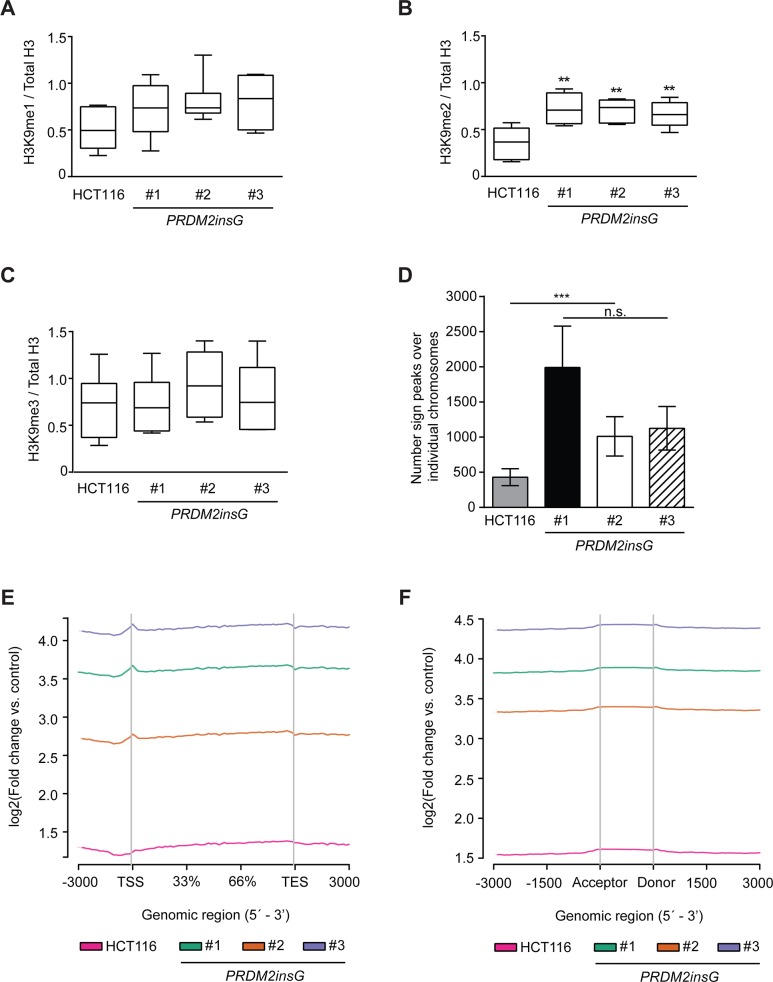
The c.4467delA mutation compromises H3K9 methyltransferase activity of *PRDM2* **(A-C)** Quantification of immunoblot images from 6 independent experiments detecting mono- (A), di- (B) and trimethylation (C) of H3K9. The signals for H3K9 methylation were normalized to total H3. ^***^ *P*<0.001, ^**^ *P*<0.01 (Mann-Whitney U test). All immunoblot images are shown in Supplementary Figure 2. **(D)** Summary of peaks detected over individual chromosomes of parental HCT116 cells and *PRDM2insG* clones. ^***^ *P*>0.001 (Student's t-test). **(E-F)** Enrichment of H3K9me2 peaks in (E) gene bodies and (F) splice sites of *PRDM2insG* clones when compared to parental HCT116 cells.

We next sought to test whether the correction of one mutant allele leads to the restoration of protein function. On the basis of previous findings suggesting PRDM2's role in methylation of H3K9, we measured the levels of global H3K9 methylation marks in parental HCT116 and *PRDM2insG* cells [19]. While the tendency towards increased H3K9me1 was not significant in *PRDM2insG* clones (Figure 2A), a significant increase of H3K9 dimethylation (H3K9me2) was observed in all three *PRDM2*-corrected clones (Figure 2B and Supplementary Figure 2). Global H3K9me3 levels were not affected by *PRMD2* correction (Figure 2C). Since H3K9me2 was the only modification that was significantly increased, we performed chromatin immunoprecipitation and deep sequencing of the associated DNA to determine where in the genome PRDM2 would place the H3K9me2 mark. We found that H3K9me2 peaks indeed increased on average three-fold at genic regions in all three clones (Figure 2D), with a sharp peak at the transcriptional start site (TSS) as compared to the parental line (Figure 2E). Exonic regions also had slight enrichment over non-exonic regions (Figure 2F). When plotting the reads delineated by the individual chromosomes, we found that the increase of H3K9me was largely global (Supplementary Figure 3). Together, these results clearly show that our gene targeting approach restored the function of PRDM2 in three independent *PRDM2insG* clones as indicated by their increased dimethylation of H3K9.

### Impact of restored PRDM2 function on cancer phenotypes

**Figure 3:**
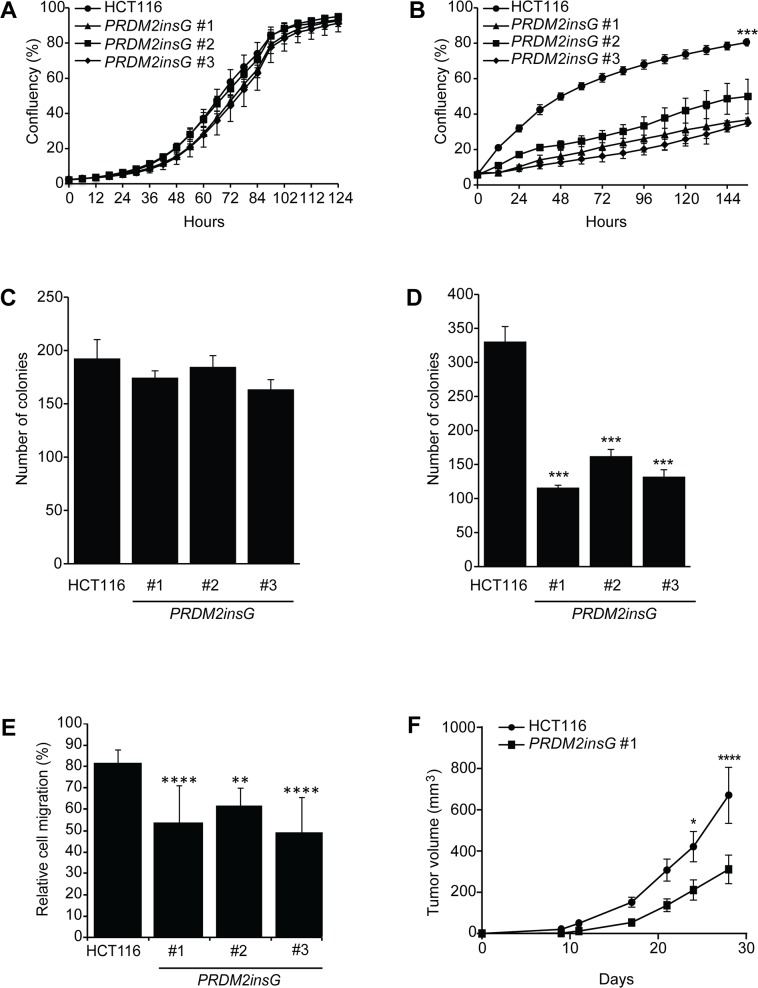
Loss of *PRDM2* increases tumorigenicity *in vitro* and *in vivo* **(A-B)** Growth rate of parental HCT116 and *PRDM2insG* cells under normal (A) and reduced serum (B) conditions. **(C-D)** Colony formation assay of parental HCT116 and *PRDM2insG* cells under normal culturing conditions in 2D cultures (C) and in soft agar (D). ^***^ *P*<0.005, Student's *t* test. **(E)** Migratory properties of *PRDM2insG* clones and HCT116 cells in a wound-healing assay. The average scratch width 72 hours after wounding is shown. ^****^ *P*<0.001, ^**^ *P*<0.01, one-way ANOVA. **(F)** The *in vivo* growth rate of parental HCT116 (*n* = 10) and *PRDM2insG*#1 cells (*n* = 8). Tumor volume was measured at the indicated time points. ^****^ *P*<0.001, ^*^ *P*<0.05, two-way ANOVA. All data are presented as mean values and SD.

As truncated PRDM2 has been suggested to have a compromised tumor suppressor function, the growth properties of the three *PRDM2insG* clones were compared to parental cells. While no difference between the PRDM2 restored clones and parental HCT116 cells was observed under normal culture conditions (Figure 3A), cells with restored PRDM2 activity showed impaired growth under reduced serum concentrations (Figure 3B). Interestingly, growth under reduced serum conditions also led to a more pronounced formation of densely packed cell clusters of *PRDM2insG* cells (Supplementary Figure 4). Restoration of PRDM2 had no influence on the two-dimensional colony-forming ability of cells under standard culture conditions (Figure 3C and Supplementary Figure 5A). In contrast, anchorage independent growth was reduced three-fold in all *PRDM2insG* clones (Figure 3D and Supplementary Figure 5B). To assess the effect of *PRDM2* restoration on the migratory capabilities of HCT116 cells we performed wound healing assays under low serum conditions. A decrease of 1.3 to 1.7-fold in migration rate was observed in all three *PRDM2insG* clones after 72 hours when compared to parental HCT116 cells (Figure 3E and Supplementary Figure 6). When the *in vivo* growth properties of *PRDM2insG* cells were tested in a mouse xenograft model, a 2-fold decrease in the growth rate of tumors with corrected *PRDM2* was revealed relative to parental HCT116 cells (Figure 3F). Thus, the restoration of one allele of *PRDM2* in HCT116 cells has a negative impact on tumor cell growth and invasiveness.

### Identification of PRDM2 regulated genes

**Figure 4:**
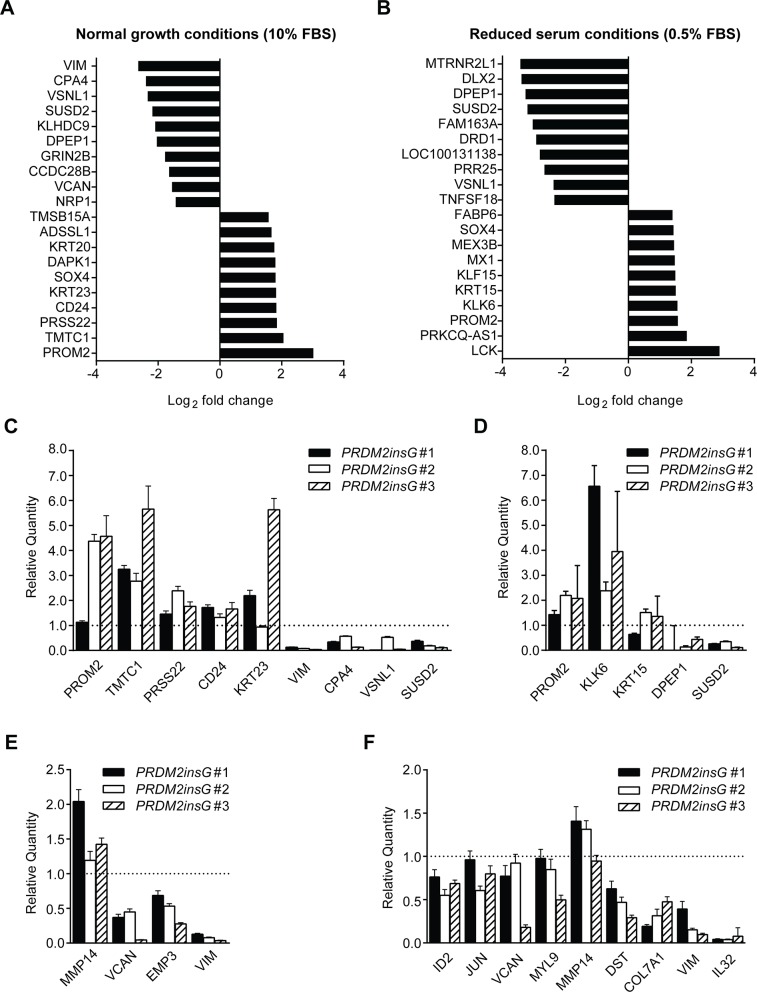
Genes significantly deregulated in HCT116 cells with corrected *PRDM2* **(A-B)** Top 10 PRDM2-deregulated genes identified under normal (A) and reduced (B) serum conditions. **(C-D)** RT-qPCR validation of PRDM2-deregulated target genes identified under normal (C) and reduced (D) serum conditions. **(E-F)** RT-qPCR validation of PRDM2-deregulated genes involved in EMT under normal (E) and reduced (F) serum conditions. Genes validated in at least two of the three clones are shown. Bars represent minimum and maximum relative quantity (RQ) calculated by the StepOne software (Applied Biosystems). The dotted line indicates the level of gene expression in parental HCT116 cells. Data from one experiment are presented.

Several studies have suggested the involvement of PRDM2 in transcriptional regulation, having either a suppressive or an activating role [20–22]. To determine to what extent the transcriptional regulation by PRDM2 is affected by C-terminal frameshift mutations, we sequenced mRNA isolated from parental HCT116 and *PRDM2insG* cells cultured under both normal and reduced serum conditions. In cells grown under normal culture conditions, 132 genes (all protein encoding) were differentially expressed 1.5-fold or more in all clones with corrected *PRDM2* compared to parental HCT116 cells (Figure 4A and Supplementary Table 5). Of these, 85 genes were downregulated and 47 were upregulated in *PRDM2insG* cells. Transcriptome analysis of cells grown under reduced serum conditions yielded 167 genes (four being noncoding RNAs) with ≥ 1.5-fold change in expression level in clones with restored PRMD2 (Figure 4B and Supplementary Table 6). Restored PRDM2 led to downregulation of 111 genes whereas 56 genes were upregulated. Comparison of differentially expressed genes between the two growth conditions revealed 18 downregulated and 15 upregulated genes found in both gene sets. Thus, a limited number of genes have their expression altered as a consequence of PRDM2 mutation in CRC cells.

### Pathway analyses and validation of differentially expressed genes

We next performed a gene enrichment analysis where genes were functionally grouped using gene ontology (GO) categories. No enriched GO terms were revealed among genes whose differential expression was observed when cells were cultured under normal conditions. In contrast, over 100 GO terms (FDR q-value < 0.05) representing broad biological processes and molecular functions were obtained when analyzing genes differentially expressed under reduced serum conditions (Supplementary Table 7). When subjected to gene set enrichment analysis based on the Hallmark gene sets [23], the five top-scoring categories (all with FDR q-value < 10^-4^) were associated with cancer-related processes such as epithelial-to-mesenchymal transition (EMT), TGF-beta signaling, TNFA signaling and KRAS signaling (Table 1). Overexpression of vimentin a marker of EMT, has been observed in several epithelial cancer types and is often associated with poor prognosis [24]. Here, *VIM* showed the most pronounced downregulation in *PRDM2insG* cells grown under normal medium conditions (6-fold decrease, q = 0.02) whereas under reduced serum a 3-fold decrease in expression (q = 0.024) was observed in these cells when compared to parental HCT116 cells. To further explore gene sets showing differential expression upon correction of *PRDM2* mutation, enrichment analysis was performed on the set of transcription factor target gene signatures available from the Molecular Signature Database (MSigDB, Supplementary Table 8). A significant enrichment for genes regulated by MAZ, LEF1, SP1, TCF3 and TAF was observed under both culture conditions indicating their potential cooperation with PRDM2. Interestingly, PRDM2 has also been identified as a transcriptional repressor binding to GC rich Sp1 elements [25]. For validation, thirty-six deregulated genes, including (1) the top five genes with the most significant up and downregulation in both growth conditions and (2) genes from the EMT set (six and ten for normal and reduced serum conditions, respectively), were subjected to RT-qPCR. RNA sequencing results were validated in 13 of 16 tested genes differentially expressed in *PRDM2insG* cells grown under normal culture conditions (Figure 4C and 4E). When analyzing genes differentially expressed under reduced serum conditions, the RT-qPCR analysis was in agreement with RNA sequencing data in 14/18 successfully amplified transcripts (Figure 4D and 4F). Taken together, the strong EMT signature, particularly in the gene expression profile from reduced serum culture, aligns with the increased tumorigenicity measured by anchorage-independent growth, *in vivo* growth, and migratory properties of HCT116 cells with truncated PRDM2. To further corroborate that the expression of genes found deregulated in *PRDM2insG* cells is indeed affected by restoration of PRDM2 we measured the level of expression of selected genes following shRNA-mediated depletion of PRDM2 in *PRDM2insG*#1. While no difference in expression was observed for *VCAN*, *EMP3*, *SUSD2*, *TMTC1*, *VIM*, *VSNL1, TNTC1, KRT23 and MMP14, CPA4* was shown to be upregulated in *PRDM2insG*#1 cells (1.6 – fold) after depletion of PRDM2 (Supplementary Figure 7). This indicates that *CPA4* expression may be directly regulated by PRDM2.

**Table 1:** Cancer pathways regulated by PRDM2

Culturecondition	Gene Set Name	# Genes inGene Set (K)	# Genes in Overlap (k)	k/K	*P*	*Q*
	EPITHELIAL_MESENCHYMAL_TRANSITION	200	6	0,03	2,30 × 10^−5^	3,83 × 10^−4^
Normal (10% FBS)	KRAS_SIGNALING_UP	200	6	0,03	2,30 × 10^−5^	3,83 × 10^−4^
	TNFA_SIGNALING_VIA_NFKB	200	6	0,03	2,30 × 10^−5^	3,83 × 10^−4^
	UV_RESPONSE_DN	144	5	0,03	5,67 × 10^−5^	5,86 × 10^−4^
	CHOLESTEROL_HOMEOSTASIS	74	4	0,05	5,86 × 10^−5^	5,86 × 10^−4^
	EPITHELIAL_MESENCHYMAL_TRANSITION	200	10	0,05	2,36 × 10^−9^	1,18 × 10^−7^
Serum starved (0.5% FBS)	TNFA_SIGNALING_VIA_NFKB	200	9	0,04	3,79 × 10^−8^	7,24 × 10^−7^
	UV_RESPONSE_DN	144	8	0,05	4,34 × 10^−8^	7,24 × 10^−7^
	TGF_BETA_SIGNALING	54	4	0,07	3,81 × 10^−5^	4,77 × 10^−4^
	KRAS_SIGNALING_UP	200	6	0,03	7,40 × 10^−5^	7,40 × 10^−4^

Hallmark gene sets were identified by gene set enrichment analysis of differentially expressed genes in *PRDM2insG* cells grown under normal and low serum culture conditions. P, hypergeometric p value; Q, P-value corrected for multiple testing.

## DISCUSSION

The tumor suppressor role of PRDM2 has previously been suggested based on several lines of evidence. In addition to its frequent inactivation in different cancer types, the increased incidence of a broad spectrum of tumors in *PRDM2*/*RIZ1* KO mice [12] and impaired tumorigenicity in nude mice following overexpression of PRDM2/RIZ1 [13] are indicative of this. While there is genetic evidence supporting *PRDM2/RIZ1* as a tumor suppressor, the functional significance of the common frameshift mutations in coding (A)8 and (A)9 repeats in cancers has not been directly demonstrated. Genome editing in human tumour cells is a preferred approach to study the phenotypes of somatic mutations. Here we have generated a stringent isogenic model where the only difference between parental HCT116 cells and the derivative cell lines is the correction of one *PRDM2* allele to wild-type sequence by insertion of one base. Using this model we demonstrate for the first time that the c.4467delA frameshift mutation in *PRDM2* leads to deregulated growth and increases the malignant potential of human tumor cells.

The importance of the PR binding motif in regulating PRDM2 activity was first shown by *in vitro* characterization of truncation mutants finding the C-terminal region necessary and sufficient for PR binding and intra- and intermolecular interactions [9]. Notably, the same C-terminal region was found to interact with both the PR domain of PRDM2 and the SET domain of PR-Set7, indicating that these C-terminal truncations may have broader effects besides modulating PRDM2 activity. While the H3K9 methyltransferase activity of PRDM2 has been shown both *in vitro* and *in vivo*, it remains unclear whether PRDM2 has monomethyltransferase, dimethyltransferase or dual activity [8, 26, 27]. Moreover, some reports provide evidence for PRDM2 acting as a global enzyme [26], whereas others show its activity to be localized to specific loci [27]. First, while G9a, a closely related H3K9 methyltransferase, targets the chromatin to place both mono and di methylation of H3K9 ubiquitously, we find that PRDM2 only places H3K9 dimethylation. Second, to shed light on genome-wide targeting of PRDM2, we performed ChIP-seq analysis of H3K9me2 which decisively showed that it acts as an H3K9 dimethylase across all chromosomes and that it largely targets genic regions with a defined peak at the TSS in all restored clones compared to the parental line. While studies have shown that G9a and its closely G9a-related protein GLP are the main enzymes placing H3K9 dimethylation at eukaryotic genes, it remains to be shown how specific or redundant they are with respect to PRDM2 [28, 29].

We show here that the correction of one c.4467delA allele to wild-type sequence has a profound effect on cancer associated phenotypes. In addition to impaired growth under low serum and reduced migration properties, all three isogenic *PRDM2insG* clones showed at least 3-fold reduced colony forming ability in soft agar. Moreover, we observed a 2-fold decrease in the *in vivo* growth rate of *PRDM2insG* xenografts, demonstrating that loss of PRDM2 contributes to a more aggressive cancer phenotype. Although this study corroborates the impaired *in vivo* growth others have observed in xenografts overexpressing PRDM2 [13], we could not detect any effect of PRDM2 correction under normal culture conditions. This is also in contrast with reports showing overexpression of full-length RIZ1 to induce G2/M arrest and/or apoptosis, which was reported even in HCT116 cells [13, 18, 30]. The major difference between our experimental model and the studies based on introduction of exogenous PRDM2/RIZ1 is that genome editing preserves physiological levels of gene expression whereas exogenous DNA is placed under the control of a strong promoter. The observed discrepancies can therefore likely be explained by the level of PRDM2/RIZ1 in cells. The RIZ2 isoform may be functionally affected by c.4467delA and some of the effects observed here may therefore be RIZ2 mediated. Taken together, our findings provide direct evidence for *PRDM2* c.4467delA as a driver mutation in MSI CRCs.

The involvement of PRDM2 in transcriptional regulation, either as a suppressor or an activator, has been suggested by several studies. Prior attempts to determine a set of genes regulated by PRDM2 include gene expression array analysis of chronic myeloid leukemia cells expressing exogenous PRDM2, revealing several genes involved in insulin-like growth factor signaling as targets [31], and expression analysis of livers from PRDM2 knockout mice identifying 97 putative PRDM2 target genes [32]. Interestingly, the sets of PRDM2-regulated genes identified through these efforts do not overlap between studies or with the genes reported here. Plausible explanations include that (1) the genetic background and tissue origin of the analysed systems may determine which processes are mediated by PRDM2, and (2) there are inherent differences between transient transfection and genome editing as experimental models. Here we show a moderate change in the gene expression profile of HCT116 cells following *PRDM2* correction. To identify potential biological processes altered through PRDM2 restoration, Gene Set Enrichment Analysis (GSEA) was performed on the set of PRDM2 deregulated targets. This revealed a significant enrichment for genes involved in EMT in *PRDM2insG* clones grown both under normal and low serum culture conditions. EMT is a reversible embryonic program that plays an essential role in several developmental processes [33]. It is frequently triggered during the progression of cancer and allows cells to gain migratory and invasive properties [34]. Several of the EMT genes identified by us as putative PRDM2 targets have been previously shown to be overexpressed in several cancer types. Examples include *VCAN*, *VIM* and *IL32*, mediators of cell motility and invasion when upregulated in ovarian cancers, HNSCCs and metastatic CRCs, respectively [35–37]. The downregulation of such genes observed here suggests that restoration of PRDM2 H3K9me2 activity leads to transcriptional repression of genes involved in EMT and diminishes the cancer associated phenotypes of parental HCT116 cells.

In summary, we have generated an isogenic cell system that allows precise characterization of the *PRDM2* c.4467delA mutation. We have presented the first direct evidence for the role of c.4459delA as a driver mutation in CRC that affects global H3K9 dimethylation and that the inactivation of PRDM2 by C-terminal truncation is linked to increased tumorigenic potential. The approach pioneered here, knock-in correction with stabilization of repeat tracts, can be applied to two-thirds of repeats mutated in MSI CRCs and should have general applications in future studies of cancer genes.

## MATERIALS AND METHODS

### Generation of PRDM2 targeting construct

The rAAV gene targeting vector used to correct the *PRDM2* mutation in human HCT116 cell line was designed to contain guanine within the (A)9repeatinstead of the deleted adenine to divide the mononucleotide repeat and thereby stabilize the sequence. The primers used for vector construction and screening are listed in Supplementary Table 1. The left and right regions of homology (HA1 containing *PRDM2insG* and HA2) were PCR amplified using HCT116 genomic DNA as a template with Phusion High-Fidelity DNA polymerase (Finnzyme) and *attB* tailed primers 1 - 4. The PCR conditions were initial denaturation at 98°C for 30 sec, 3 cycles of denaturation at 98°C for 10 sec, annealing at 61°C for 15 sec and extension at 72°C for 30 sec followed by three cycles at 58°C and 55°C annealing temperature, respectively. The final amplification had 25 cycles of denaturation at 98°C for 20 sec, annealing at 54°C for 15 sec and extension at 72°C for 30 sec. To generate the targeting construct both HAs and selection cassette were cloned into the destination vector using the Gateway system as described in Stoimenov *et al* [17]. Briefly, 100 ng of the amplified HA1 and HA2 were recombined with 150 ng of pDONR^TM^P1-P2 and pDONR^TM^P3-P4 using BP Clonase II (Invitrogen) and the resulting clones were tested in PCR reaction with M13 primers 5 - 6. In order to insert one G within the (A)9 repeat of pDONR^TM^P1-P2 containing HA1, site-directed mutagenesis was performed using the QuickChange II mutagenesis kit (Stratagene) with primers 7 - 8 and the insertion was confirmed by Sanger sequencing. In the next step, 10 fmol of each pEntry-HA1, pBUOY.SA.IRES.Neo.pA and pEntry-HA2 were recombined with AAV-Dest vector using LR Clonase II (Invitrogen). The presence and orientation of fragments was confirmed by PCR and Sanger sequencing using LR screening primers 9 - 12. To produce virus particles, AAV-293 cells were grown in DMEM supplemented with 10% FBS and 1% penicillin/streptomycin (all from Gibco/Life Technologies). At 70% confluency, the cells were transfected with 5 μg each of plasmids pAAV-RC, pHELPER (Stratagene) and the targeting construct in 1.5 ml of OptiMEM and 54 μl Lipofectamine (Invitrogen). The viral particles were harvested as crude cell lysate 48 hours after transfection by performing three freeze-thaw cycles between a dry-ice methanol bath and a 37°C water bath followed by collection of the virus-containing supernatant after centrifugation.

### Gene targeting in HCT116 cells

The human colorectal cancer cell line HCT116 (ATCC, obtained in 2008) was maintained in McCoy's 5A medium supplemented with 10% FBS and 1% PEST (all from Gibco/Life Technologies. Following transfection with the *PRDM2insG* targeting vector cells were plated in 96-well plates at limiting dilution and grown in the presence of 0.4 mg/ml Geneticin (Gibco/Life Technologies) for two weeks. Site-specific integration of the targeting vector in Geneticin-resistant single-cell clones was confirmed by PCR using primers 13 and 14 for HA1 and primers 11 and 15 for HA2. The integrity of sequence surrounding the integration site within exon 8 of *PRDM2* as well as the presence of inserted G was assessed by Sanger sequencing using primers 13 and 16. Three independent clones with corrected *PRDM2* allele were expanded and the drug resistance cassette was removed by infection with Ad-Cre virus (Vector Biolabs). Single-cell clones that were obtained after plating at limiting dilution were screened by PCR using primers 17 - 18 in order to identify those where the Neo marker was removed. In addition, positive clones were verified by their inability to grow under selection with 0.4 mg/ml Geneticin. To verify the expression of the corrected *PRDM2* allele, total RNA was extracted from the three clones with RNeasy Plus Mini kit (Qiagen) and cDNA was synthesized using Maxima H Minus First Strand cDNA Synthesis kit (Thermo Scientific). RT-PCR products generated with primers 19 - 20 were subsequently Sanger sequenced. HCT116 parental cells and clones were authenticated in 2016 by STR analysis (Promega PowerPlex 18D, ATCC) and tested for Mycoplasma using the MycoAlert mycoplasma detection kit (Lonza). Cells were kept at a low passage number between thawing and the conduction of the different experiments.

Design of rAAV gene targeting constructs for correction and stabilization of repeats in genes frequently subject to indel mutations in MSI CRCs. Twenty-five repeats observed mutated in TCGA COAD [16] were subject to automatic design of rAAV gene targeting constructs using software, design criteria and construct selection criteria described in [17]. The suggested PCR primers for generating rAAV constructs for targeting 18 of the repeats are presented in Supplementary Table 2.

### Cell growth rate, colony formation assay and wound healing

Cells were plated in triplicate wells at a density of 20,000 cells per well in 24-well plates. The next day, the growth medium was changed to either 10 % FBS or 0.5 % FBS and the cells were imaged in real time with an IncuCyte HD instrument (Essen BioScience) for growth rate analysis. For colony formation analysis, 500 cells per well were plated in 6-well plates in triplicates. Colonies were stained with 5 % methylene blue in methanol and counted 10 days after seeding. To assess anchorage independent growth, 1×10^3^ cells were resuspended in 1 ml of 0.4 % top agarose (Agarose, low gelling temperature A9414, Sigma Aldrich) and overlaid onto 1 ml of 0.8 % solid bottom agarose in each well of 6-well plates in triplicates. After solidification, the top agarose was covered with 1 ml of culture medium. Cells were incubated for 2 – 3 weeks and colonies were visualized by staining in 0.05 % crystal violet. For wound healing assays, cells were plated in triplicate wells at a density of 300,000 cells per well in 12-well plates and grown under normal (10 % FBS) growth medium conditions. When confluent, a scratch was made on each well using a 10 μL pipette tip. Free-floating cells were subsequently removed by washing with HBSS and growth medium with low serum content (0.5 % FBS) was added. Cells were observed using a light microscope with 10× magnification at 0 h and 72 h. Photomicrographs of different sections of the scratch were taken for each well and for each time point using the NIS-Elements Ar Imaging Software (Nikon). To estimate the relative cell migration of parental HCT116 cells and each of the three *PRDM2insG* clones, the average scratch width was calculated for each clone at 0 h and 72 h using the image processing program ImageJ (http://imagej.nih.gov/ij/).

### Xenograft growth assay

Animal experiments were performed in compliance with institutional guidelines and approved by the local animal ethical committee in Uppsala, Sweden (C55-2014). Eight to nine weeks old female NMRI Nu/Nu mice (Taconic) were subcutaneously inoculated with 2.5 × 10^6^ parental HCT116 cells or 2.5 × 10^6^ *PRDM2* corrected cells. Tumor size was measured until the volume of xenografts from HCT116 cells reached 1 cm^3^.

### RNA sequencing

RNA was extracted from cells cultured under both normal culture and reduced serum (0.5%) conditions. RNA samples were prepared as described above. The RNA integrity and concentration was determined on a Bioanalyzer instrument (Agilent) using an RNA 6000 Nano kit. Samples were sequenced on the Ion Proton system (Life Technologies). In each sample, 30-50×10^6^ reads were sequenced (NCBI GEO accession number GSE80033). The reads were aligned to the human genome hg19 assembly (https://genome.ucsc.edu) using Tophat2 software (version 2.0.4) [38]. The read count quantification and differential expression analysis were performed using the Cufflinks tool (version 2.2.1) [39]. GSEA was performed using the MSigDB collection of annotated sets of genes (http://www.broadinstitute.org/gsea/msigdb/).

### Real-time quantitative PCR

Total RNA was extracted and cDNA prepared as described above. RT-qPCR was performed with Maxima SYBR Green/Rox qPCR Master Mix (Thermo Scientific) in an StepOne qPCR instrument (Applied Biosystems) with primers listed in Supplementary Table 3. Data were analyzed by the ΔΔCt method with the StepOne^TM^ Software 2 using *PGK*, *TBP* and *ACTB* as reference genes.

### shRNA depletion of PRDM2

The pGIPZ lentiviral shRNA vector targeting PRDM2 (RHS4430-200269018) as well as a non-silencing pGIPZ control vector (RHS4346), both from Thermo Scientific, were used to transiently transfect *PRDM2insG* clone 1 and HCT116 cells. Cells were seeded in 6–well plates and transfection was performed with Lipofectamine 2000 (Life Technologies) using 5 μg of recombinant construct. Cells were harvested 48 h post transfection and RNA was extracted and cDNA synthesized as previously described. RT-qPCR for *VCAN*, *EMP3*, *CPA4*, *VIM*, *VSNL1*, *SUSD2, KRT23, MMP14* and *TMTC1* was carried out as mentioned above.

### Western blot

Histone extracts were prepared from 2×10^6^cells using the Histone Extraction Kit (Abcam, ab113476). Equal amounts of proteins were separated on 4-12% SDS-PAGE, transferred onto nitrocellulose membranes and probed with anti-H3K9me1 (ab9045, Abcam; 1:2000), anti-H3K9me2 (ab1220, Abcam; 1:1000), anti-H3K9me3 (ab8898, Abcam; 1:2000), or anti-H3 (ab1791; 1:4000). Immunoreactive proteins were visualized using SuperSignal West Femto Chemiluminescent Substrate (Thermo Scientific) on the ImageQuant LAS 4000 imaging system (GE Healthcare). The ratio of methylated H3K9 to total H3 was quantified by densitometric analysis of Western blots using the image processing program ImageJ (http://imagej.nih.gov/ij/).

### Chromatin immunoprecipitation

Chromatin immunoprecipitations were performed as previously described [40]. In brief, 10 × 10^6^ parental HCT116 cells and restored *PRDM2insG* clones were seeded in Ø15 cm plates and crosslinked with 1% formaldehyde (ThermoScientific), quenched with 100 mM glycine, rinsed with PBS and collected with cell scrapers. Nuclei were purified with mild sonication using Covaris S220 followed by shearing to 100-800 bp in protease inhibitor-containing lysis buffer, according to the Nexon protocol [41]. Sheared chromatins were precipitated with the H3K9me2 antibody ab1220 (Abcam), reversed cross-linked, proteinase K-treated and DNA purified.

### ChIP library and deep sequencing

The immunoprecipitated and purified HCT116 genomic DNA, including the input DNA as control, were used for library preparation with Illumina TrueSeq reagents. To verify the size of PCR enriched fragments, the template size distribution was analyzed on a DNA1000 chip in a 2100 Bioanalyzer (Agilent Technologies). Sequencing reads were generated on the Illumina HiSeq2500 platform. Single-end raw reads were trimmed (Q30), mapped (BWA read mapping), and subject to duplicate reads removal (Picard), peak calling (MACS), and peak annotation (SnpEff) for downstream analysis.

## SUPPLEMENTARY MATERIALS FIGURES AND TABLES



















## Abbreviations

BWA: Burrows-Wheeler Aligner
COAD: colon adenocarcinoma
CRC: colorectal cancer
DMEM: Dulbecco's Modified Eagle's Medium
EMT: epithelial-to-mesenchymal transition
FBS: fetal bovine serum
FDR: false discovery rate
gDNA: genomic DNA
GO terms: gene ontology terms
GSEA: Gene Set Enrichment Analysis
HA: homology arm
MACS: Model-based Analysis of ChIP-Seq
MMR: mismatch repair
MSI: microsatellite instability
MSigDB: Molecular Signature Database
PRDM2: PR domain zinc finger protein 2
rAAV: recombinant adeno-associated virus
RT-qPCR: quantitative reverse transcription PCR
shRNA: short hairpin RNA
TCGA: The Cancer Genome Atlas
TNF signaling: tumor necrosis factor signalling.
