# Supplementary material for: Somatic PRDM2 c.4467delA mutations in colorectal cancers control histone methylation and tumor growth
Source: Oncotarget. 2017 Oct 9;8(58):98646–59. doi: 10.18632/oncotarget.21713 (PMC5716757; doi:10.18632/oncotarget.21713)
Supplement: Supplementary file 1 [file oncotarget-08-98646-s001.pdf]

Somatic *PRDM2* c.4467delA mutations in colorectal cancers control histone methylation and tumor growth

SUPPLEMENTARY MATERIALS

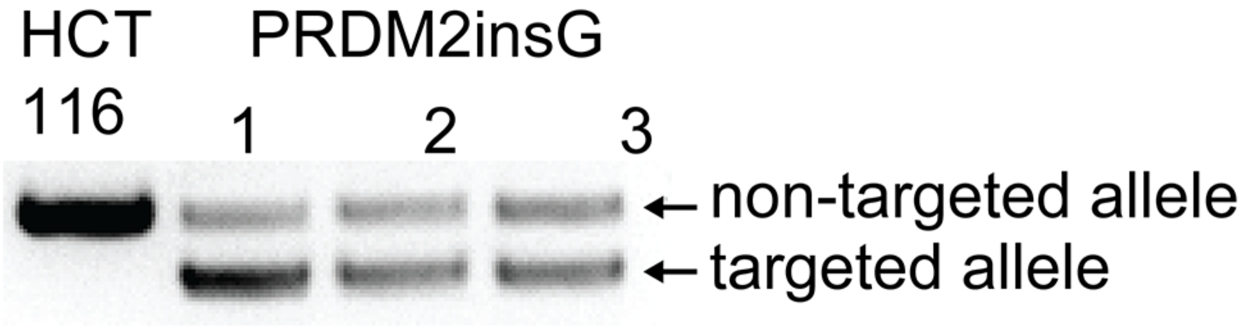

Supplementary Figure 1: Excision of selection cassette in targeted *PRDM2insG* cells. Successful excision of the neo selection cassette following *Cre*-mediated recombination was identified by genomic PCR using primers flanking the deletion generated by correct targeting. Three independent clones are shown. Whereas a 695-bp PCR product (upper band) is generated from non-targeted allele a 529-bp PCR product (lower band) is only obtained after correct targeting.

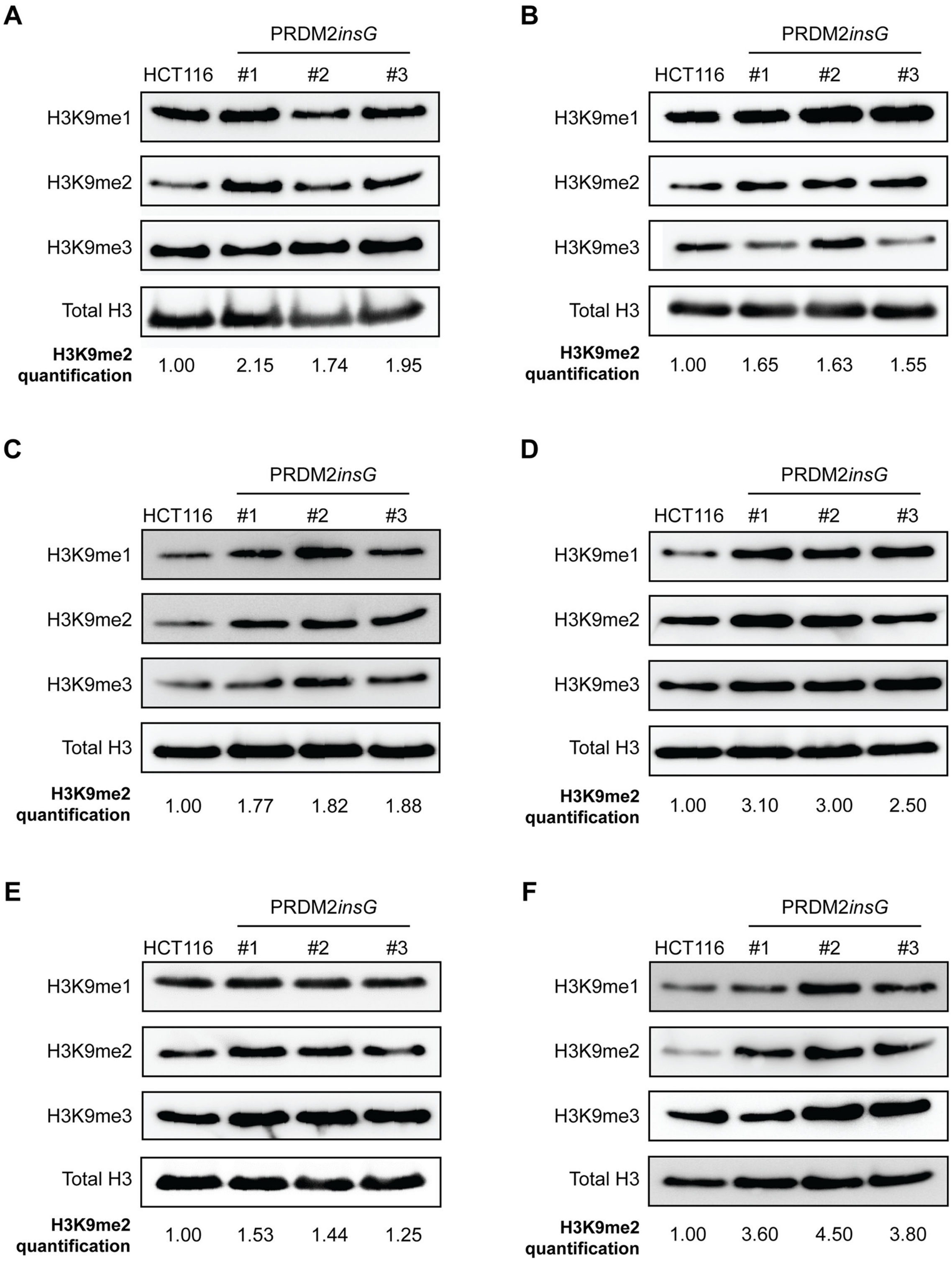

Supplementary Figure 2: The c.4459delA mutation decreases methylation of H3K9 in HCT116 cells. (A-F) Immunoblot images from six independent experiments detecting mono-, di- and trimethylation of H3K9. Quantification for H3K9me2 (normalized to total H3) is also shown.

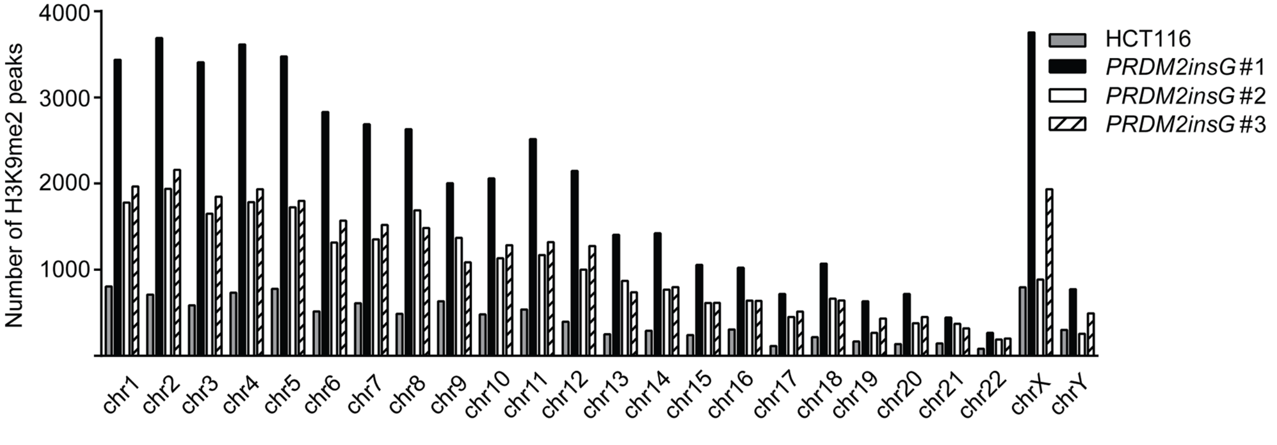

Supplementary Figure 3: Number of H3K9me2 peaks distributed across chromosomes for parental HCT116 cells and *PRDM2insG* clones.

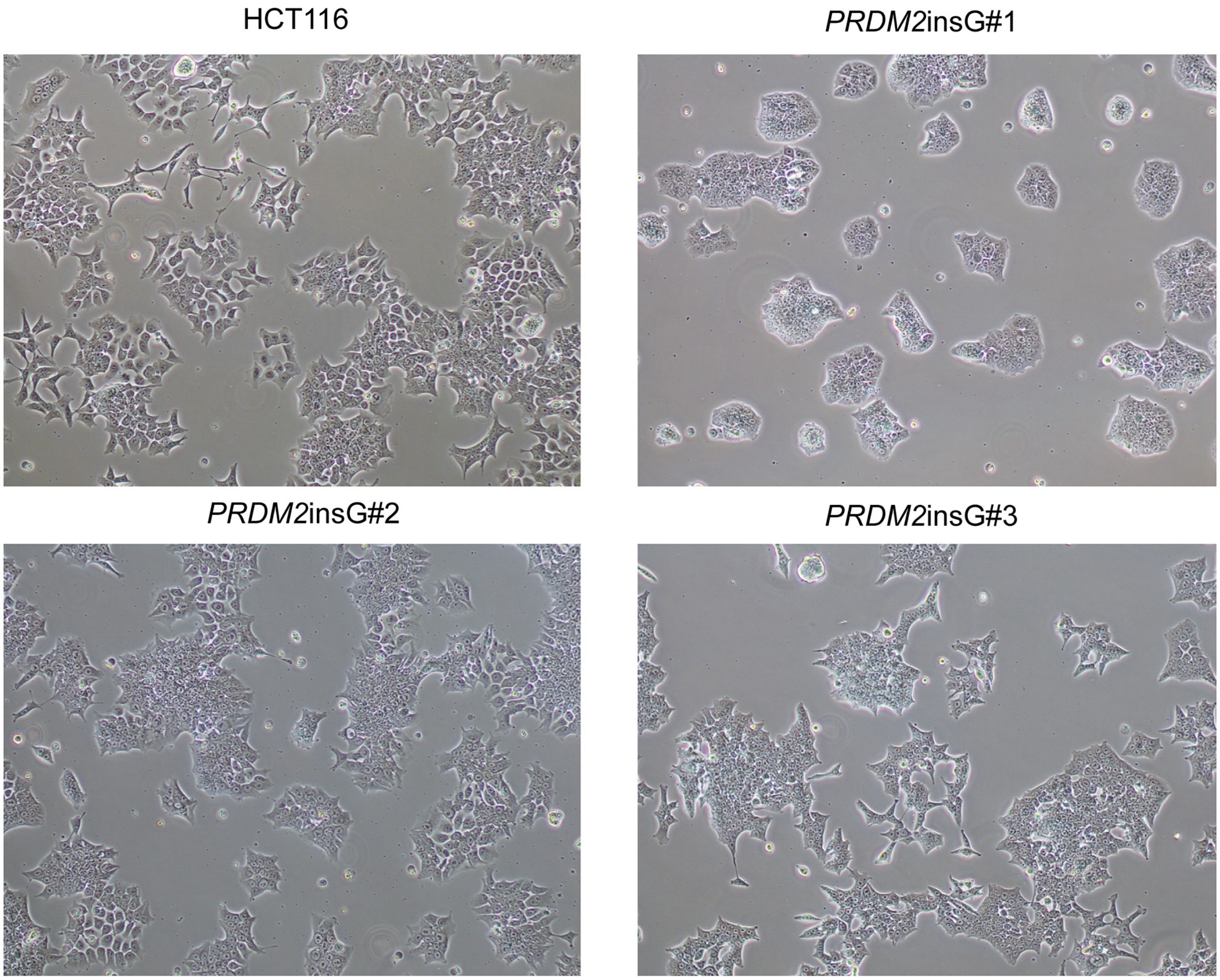

Supplementary Figure 4: Altered cellular growth pattern after correction of PRDM2. Parental and PRDM2 restored HCT116 cells were imaged at 20× magnification.

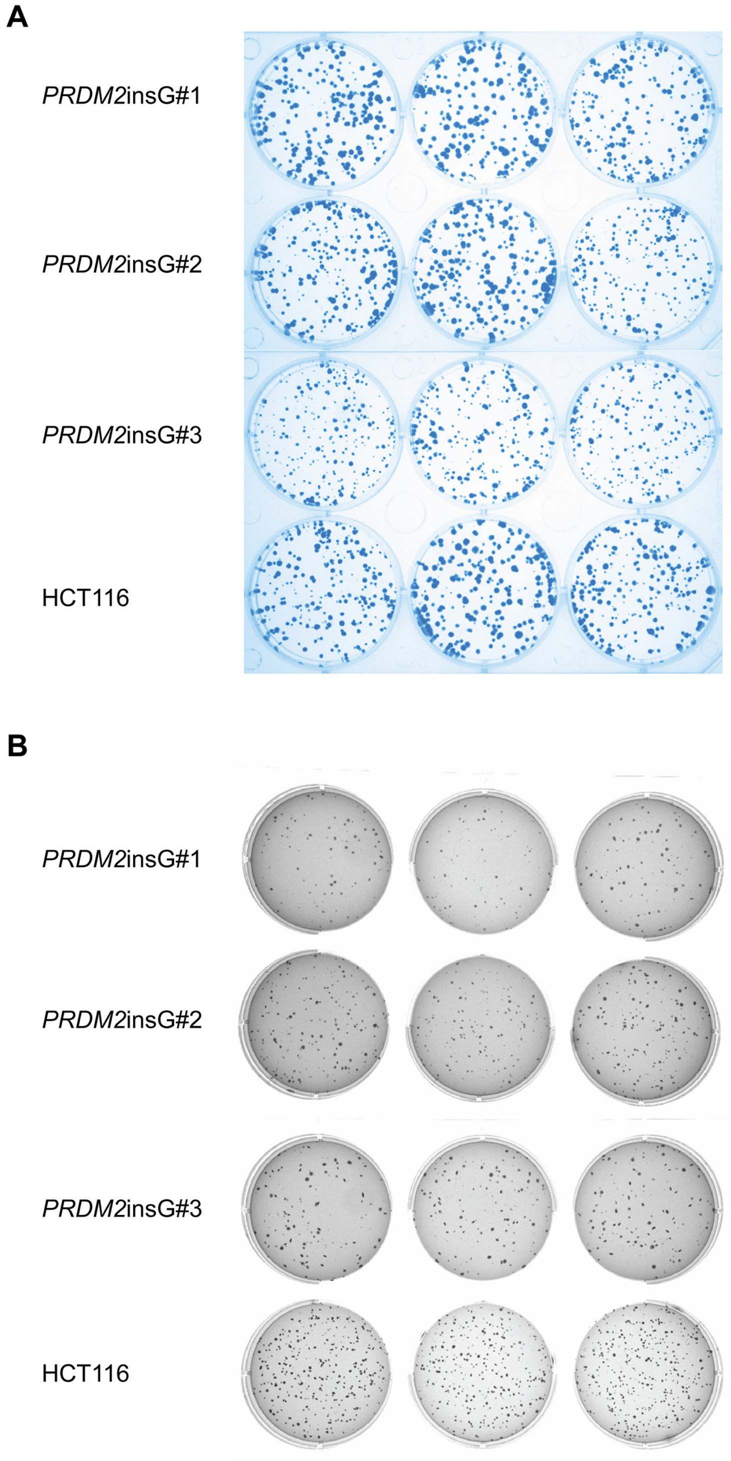

Supplementary Figure 5: Restoration of PRDM2 influences anchorage independent growth but not colony-forming ability of clones. (A) Colony formation assay. Cells were seeded in triplicates and stained after 10 days with 5% methylene blue for quantification. **(B)** Anchorage independent growth. Cells were resuspended and overlaid onto agarose in triplicates and incubated for up to 3 weeks. Cells were quantified after staining with 0.05% crystal violet.

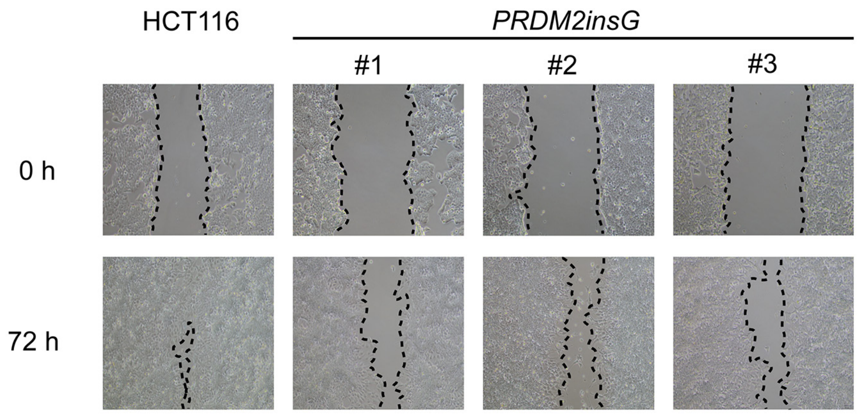

Supplementary Figure 6: Reduced migration properties of *PRDM2insG* cells revealed by wound healing assay. Representative images of wounds at 0 and 72 hours after wounding are shown. The lines indicate the wound edge at time point 0.

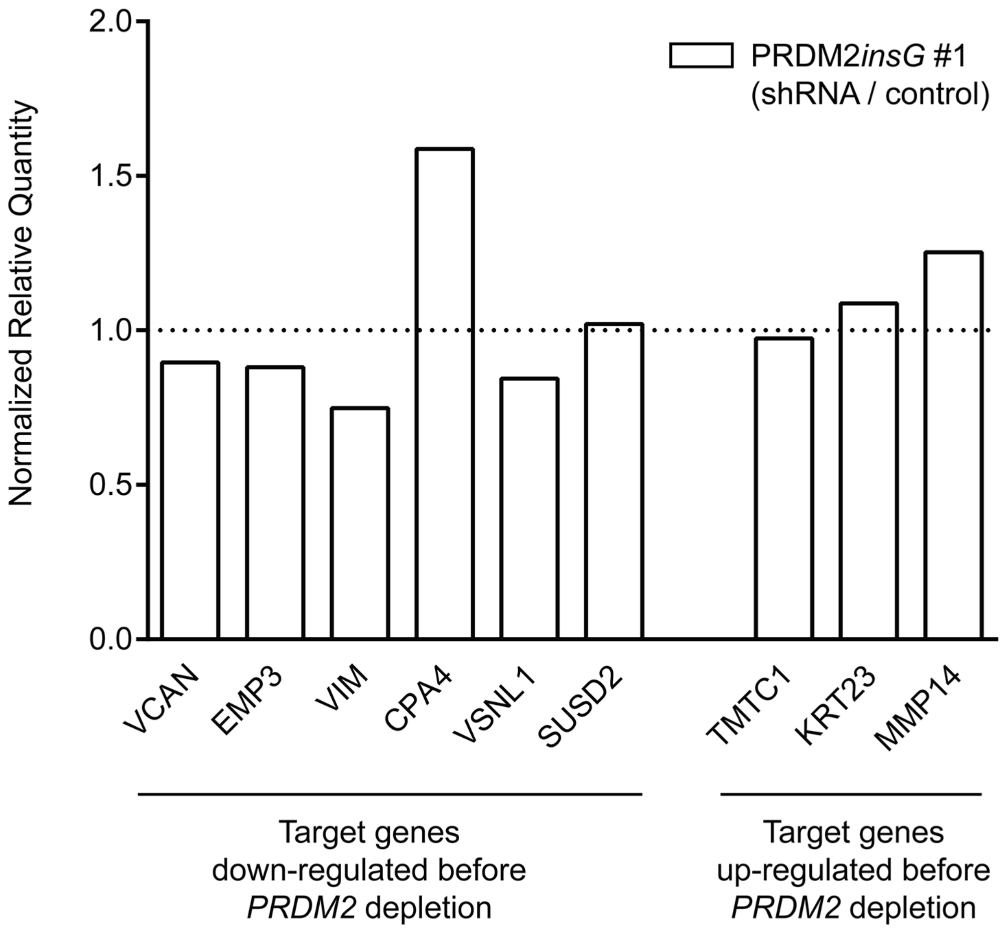

Supplementary Figure 7: RT-qPCR analysis of genes found deregulated in *PRDM2insG* clones following shRNA-mediated depletion of *PRDM2*. HCT116 parental cells and clone *PRDM2insG*#1 were subjected to analysis. HCT116 and HCT116 + shPRMD2 cells were used as controls for *PRDM2insG*#1 *and PRDM2insG*#1 + shPRDM2 cells, respectively. Y – axis shows the ratio of relative quantity values between *PRDM2insG*#1 + shPRDM2 and *PRDM2insG*#1 cells.

Supplementary Table 1: Primer sequences used for generation of isogenic *PRDM2insG* cells

See Supplementary File 1

Supplementary Table 2: Recombinant AAV homology arm designs for correction by gene targeting of MSI frameshift mutations. Repeats recurrently found mutated in TCGA COAD (16) were subjected to automated generation of rAAV gene targeting constructs. Primers for PCR amplification of homology arms of the best construct design are shown. Genome coordinates are based on human genome hg19 assembly.

See Supplementary File 2

Supplementary Table 3: Primer sequences used in RT-qPCR

See Supplementary File 3

Supplementary Table 4: Cell lines genotyped for (A)9 tract mutations in *PRDM2*. The mutation status of the (A)9 repeat harbouring c.4459delA was obtained from literature or by genotyping. [a], data from (39); [b], DNA provided by S. Markowitz and genotyped by Sanger sequencing.

See Supplementary File 4

Supplementary Table 5: Significantly deregulated genes in *PRDM2insG* cells grown under normal culture conditions. Genes that had at least 1.5 fold change in expression and false discovery rate < 5 % were included.

See Supplementary File 5

Supplementary Table 6: Significantly deregulated genes in *PRDM2insG* cells grown under reduced serum conditions. Genes that had at least 1.5-fold change in expression and false discovery rate < 5 % are shown.

See Supplementary File 6

Supplementary Table 7: Top ten GO categories identified by GSEA of genes differentially expressed in *PRDM2insG* cells grown under reduced serum conditions. Enrichment analysis was performed using the "GO" gene sets from the MSigDB (Broad Institute). *P*, hypergeometric p value; *Q*, P-value corrected for multiple testing.

See Supplementary File 7

Supplementary Table 8: Transcription factor motifs identified by GSEA of differentially expressed genes in *PRDM2insG* cells. Enrichment analysis was performed using the "Transcription factor targets" gene sets from the MSigDB (Broad Institute). *Q*-value of 10^{-6} was used as a cut off. *P*, hypergeometric p value; *Q*, P-value corrected for multiple testing.

See Supplementary File 8
